# Molecular Insights into *Rhodococcus* sp. A17: Physiological Adaptations and Degradation Characteristics for Organic Contamination at Alkaline pH

**DOI:** 10.3390/life16020252

**Published:** 2026-02-02

**Authors:** Xinyuan Wei, Haoyu Wang, Rui Li, Shengmin Liu, Hongyan Zuo, Qing Hu, Xuliang Zhuang, Zhihui Bai

**Affiliations:** 1 Research Center for Eco-Environmental Sciences, Chinese Academy of Sciences, Beijing 100085, China; weixinyuan20@163.com (X.W.); bqt1800302025@student.cumtb.edu.cn (H.W.); ruili_st@rcees.ac.cn (R.L.); xlzhuang@rcees.ac.cn (X.Z.); 2 University of Chinese Academy of Sciences, Beijing 100049, China; 3 Xiong’an Institute of Innovation, Chinese Academy of Sciences, Baoding 071700, China; 4 Dingzhou Fuyuan Food Co., Ltd., Dingzhou 073000, China; dzfysp1@163.com; 5 State Key Laboratory of Lunar and Planetary Sciences, Macau University of Science and Technology, Taipa 999078, Macau; hyzuo@must.edu.mo; 6 Institute of Tibetan Plateau Research, Chinese Academy of Sciences, Beijing 100101, China

**Keywords:** petroleum hydrocarbons, alkane degradation, degrading bacteria, bioaugmentation, alkaline pH

## Abstract

Petroleum contamination poses a serious threat to human health and ecosystems worldwide, and microbially driven natural attenuation is an effective approach for accelerating hydrocarbon removal. Species of the genus *Rhodococcus* are recognized for their ability to degrade long chain petroleum hydrocarbons. However, their physiological traits and degradation mechanisms under alkaline conditions remain insufficiently understood. In this study, soil samples were collected from the Dagang oilfield in Tianjin, China, and *Rhodococcus* sp. A17 was isolated as an active indigenous strain for genomic and physiological characterization under high pH petroleum degradation conditions. The results showed that strain A17 grew optimally at 30 °C, pH 9.0, and 2% salinity. Petroleum hydrocarbon degradation reached 67.8% within 72 h, with a degradation half life of 34.2 h. Genome sequencing identified 18 oxygenase related genes involved in alkane degradation, including *alkB*, cytochrome P450 monooxygenases, and the long chain alkane monooxygenase *ladA*, together with four antibiotic resistance genes. Metabolite analysis suggested that alkane degradation might proceed via terminal and subterminal oxidation pathways. Overall, these findings indicate that *Rhodococcus* sp. A17 exhibits multiple adaptive traits that support its potential application in the bioremediation of petroleum contaminated alkaline environments.

## 1. Introduction

Petroleum is a mixture of highly hydrophobic alkanes, cycloalkanes, and aromatic hydrocarbons, which are notoriously challenging to degrade in their natural state [1]. These compounds can enter the environment through various means, including oil spills, leakages from storage tanks and pipelines, and runoff from petrochemical production facilities [2,3]. The soil environment is negatively impacted by petroleum pollutants, especially long-chain alkanes and polycyclic aromatic hydrocarbons, which are both persistent and toxic in nature [4]. Additionally, even short-chain alkanes within petroleum have toxic effects, as they have the ability to disrupt cell membranes and impact microbial metabolism [5]. The presence of petroleum hydrocarbon pollutants in soil can result in long-lasting impacts on soil quality, function, human health, and food quality, thus constituting a significant global environmental issue that has garnered widespread public attention in recent decades [6,7].

Bioremediation is a highly desirable strategy for remediation of petroleum pollution, offering cost-effective and practical solutions [5]. This method harnesses the power of microbes to degrade hydrocarbons in contaminated sites [8]. The approach also prevents the formation of toxic byproducts, making it a more environmentally friendly and sustainable alternative to physical and chemical treatments [9]. Both indigenous and acclimated microbes can be employed to degrade petroleum contaminants [10]. For instance, Shen et al. introduced *Burkholderia cepacian* GS3C, *Sphingomonas* GY2B, and *Pandoraea* pnomenusa GP3B to petroleum-polluted soil, yielding a 64.4% reduction in total petroleum hydrocarbon after 40 days [11]. However, the efficacy of this biological approach is dependent on the survival and metabolic activity of microbes, which can be influenced by a range of physical, chemical, and ecological factors such as nutrient availability, toxicity, and competition [12,13]. Therefore, the rapid acclimation of microbial agents to the environment is crucial for successful bioremediation.

Many strains of microorganisms are renowned for their capability to efficiently endure and break down toxic compounds, including but not limited to *Halomonas* [14], *Corynebacterium* [15], *Pseudomonas* [16], *Bacillus* [17], *Acinetobacter* [18], *Microbacterium* [19], *Shewanella* [20], and *Rhodococcus* [21]. Research efforts have uncovered various elements that may impact the strains’ resilience in harsh conditions, such as cell wall toughness, metabolic flexibility, and the capability to regulate internal pH. Despite these findings, the enigma of why specific microorganisms can rapidly acclimate to high levels of petroleum contamination in alkaline pH and the mechanism behind these phenomena still persists.

In this study, soil contaminated with petroleum hydrocarbons was collected from Dagang oilfield in Tianjin, China and used to initiate a microbial consortium with petroleum as the sole carbon source. Analysis of the microbial community structure and degradation ability showed a directional effect of the pollutants on the degradation microorganisms, leading to a constant shift in community structure and enrichment of petroleum-degrading microorganisms. One notable petroleum-degrading bacterium was identified as *Rhodococcus* sp. A17. The genomic and physiological features of this model microorganism on its fast adaptation to high content of petroleum at alkaline pH were elucidated.

## 2. Materials and Methods

### 2.1. Culture Media and Chemicals

Luria–Bertani (LB) medium and minimal salt medium (MSM) were prepared according to the standard protocols [22]. Briefly, LB medium (g/L) was prepared as yeast extract 5 g, tryptone 10 g, NaCl 10 g, sterilized at 121 °C for 20 min. MSM medium (g/L) was prepared as K_2_HPO_4_ 1.5 g, KH_2_PO_4_ 0.6 g, MgSO_4_·7H_2_O 0.2 g, CaCl_2_·2H_2_O 0.1 g, FeCl_3_·6H_2_O 0.01 g, NH_4_NO_3_ 2.0 g, trace elements 1 mL, sterilized at 121 °C for 20 min. Trace elements: MoO_3_ 4 g, ZnSO_4_·5H_2_O 28 g, CuSO_4_·5H_2_O 2 g, H_3_BO_3_ 4 g, MnSO_4_·5H_2_O 4 g, CoCl_2_·6H_2_O 4 g. All reagents were purchased from Aladdin Bio-Chem Technology Co., Ltd. (Shanghai, China). The MSM formulation contained potassium salts as buffering components, while sodium salts were not included as basal constituents. A petroleum mixture with a broad carbon chain distribution (approximately C10-C35) was used as the sole carbon source and prepared by blending equal volume of NO.0 diesel fuel (China Petroleum and Chemical Corporation, Beijing, China) and engine oil (SAE 20W-40, Exxon Mobil Corporation, Tianjin, China). The MSM-petroleum hydrocarbon medium was prepared as follows: the petroleum mixture was aseptically filtered and blended into 100 mL autoclaved MSM medium in a 250 mL sterilized conical flask (GG-17, 250 mL, Sichuan Shubo Co., Ltd., Chongzhou, China). A total of 1 g of the petroleum mixture was added per flask, determined by weight using a digital balance (SE-600-1F, Shanghai Ohaus Instruments Co., Ltd., Shanghai, China). The pH of the medium was adjusted using HCl or NaOH prior to incubation; therefore, sodium ions were introduced into the medium under alkaline conditions due to NaOH addition. Unless otherwise stated, the pH of the MSM–petroleum hydrocarbon medium was adjusted to 9.0 using NaOH (3 mol L^−1^).

### 2.2. Petroleum-Degrading Consortium Enrichment and Microbial Community Profiling During the Incubation

Soil samples were collected from a petroleum contaminated soil profile at a depth of 0–20 cm in Dagang oilfield, Tianjin, China. Dagang oilfield, located near the Bohai Sea, is characterized by wetlands, rivers, farmland, and oil infrastructure. The soil profile, located at 38°69′20″ N, 117°48′03″ E, is within the oilfield and surrounded by agricultural lands. The collected soil exhibited an alkaline pH (8.86) and a total petroleum hydrocarbon concentration of 18.18 g kg^−1^. The exact duration of petroleum hydrocarbon contamination at the sampling site could not be precisely determined, but the soil has been continuously exposed to petroleum contamination in recent years. To obtain microbial colonies capable of effectively degrading petroleum hydrocarbons, about 10 g of the soil sample was added to the MSM-petroleum hydrocarbon medium. The incubation was conducted at 30 °C with 150 r/min shaking. To impose sustained selective pressure and promote microbial adaptation, the enrichment culture was periodically transferred every 14 days by inoculating 10 mL of culture into 100 mL of fresh MSM–petroleum hydrocarbon medium, over a total enrichment period of 140 days [23]. The culture was sampled six times (0, 1st, 3rd, 5th, 7th, and 10th days) in the last 14-day round of the incubation for microbial community profiling. The experiment was conducted in triplicate with one blank control which was no soil amendment. The culture DNA was extracted using the FastDNA^®^ SPIN Kit for Soil (MP Biomedicals, Santa Ana, CA, USA) according to the instructions. The integrity of the extracted DNA was examined by agarose gel electrophoresis using 1% agarose gel electrophoresis at 5 V/cm for 20 min. Miseq high-throughput sequencing was performed by Guangdong Meige Gene Technology Co., Ltd. (Guangzhou, China). Quality control of the raw sequenced sequences was performed by using fastp software (v.0.20.0) [24]. FLASHsoftware (v.1.2.7) was used for merging of paired-end reads [25]. OTU clustering of sequences based on 97% imilarity was achieved by using UPARSE (v.7.1) [26]. Species classification annotation of each sequence was conducted by using Usearch software (v11) against the Silva 16S rRNA gene database (v138) with a comparison threshold of 80% [27]. Shared and endemic species statistics, community composition analysis, and species abundance clustering analysis were performed using R software (v.4.4.0).

### 2.3. Petroleum Degradation Potential of the Enriched Consortium

About 1 mL of the enriched consortium was added to 10 mL of fresh MSM-petroleum hydrocarbon medium containing the petroleum mixture at the concentration of 10, 20, and 30 mg/mL, respectively. The incubation was conducted at 30 °C with 150 r/min shaking for 14 days. The petroleum hydrocarbons were extracted three times by using hexane (AR, Shanghai Aladdin Bio-Chem Technology Co., Shanghai, China). For each extraction, 5 mL of the culture was mixed with 5 mL of hexane. The extracts were combined and concentrated to 5 mL via rotary evaporation. The chemical composition of the extract was analyzed using a gas chromatography-mass spectrometry (GCMS-QP2010 Ultra, Shimadzu) [28] equipped with an HP-5MS silica capillary column (30 m × 0.25 mm × 0.25 μm) after filtration through a 0.22 μm organic phase filter membrane (B-GLQ22Y-13, Changde Bkmam Biotechnology Co., Ltd., Changde, China). Helium was used as the carrier gas at a flow rate of 1 mL/min. The sample was injected into the device by using a non-split injection mode with an injection volume of 1 μL and autosampler injection. The temperatures of the injection port, ion source, and interface were 280 °C, 250 °C, and 270 °C, respectively. The ramp-up procedure was 40 °C held for 2 min. Full scan mode of the mass spectrometry in the range of 24~490 m/z was applied. The real-time concentration and the depletion rate of petroleum hydrocarbons were calculated according to a standard curve prepared by plotting the peak area of the sample against the concentration of petroleum hydrocarbons.

### 2.4. Isolation, Identification, and Whole Genome Sequencing of the Petroleum-Degrading Bacterium

After three rounds of enrichment, a single bacterial colony A17 was screened and isolated. Substrate utilization tests revealed that the isolate could utilize petroleum hydrocarbons as the sole carbon source for growth and the degradation rate at pH 9 was determined. Partial 16S rRNA gene sequencing was carried out to identify the phylogeny of the bacterium. Cells were prepared for morphology observation by a scanning electron microscopy (SEM, S3400II, Hitachi Limited, Tokyo, Japan) [29]. Briefly, 1 mL LB culture of A17 cells at the exponential phase was centrifuged for 5 min at 6000 r/min and 4 °C. Afterwards, a 2.5% glutaraldehyde solution was applied to fix the cells for 12 h at 4 °C. Cells were washed three times using 0.2 M PBS (phosphate-buffered saline, pH6.8). The organisms were then sequentially dehydrated with 50%, 70%, and 100% ethanol, followed by air drying and gold plating before SEM observation. Genomic DNA from strain A17 was extracted using the FastDNA^®^ SPIN Kit (MP Biomedicals, Santa Ana, CA, USA). The V3-V4 region of the 16S rRNA gene was subsequently amplified with primers 338F (5′-ACTCCTACGGGAGGCAGCAG-3′) and 806R (5′-GACTACHVGGGTWTCTAAT-3′) and sequenced by Guangdong Meige Gene Technology Co., Ltd. (Guangzhou, China). The similarity of the sequenced partial 16S rRNA gene against to other known 16S rRNA gene sequences was evaluated using BLAST (v.2.14.0) [30]. Phylogenetic analysis of strain A17 was performed using MEGA 5.2.2, employing the neighbor-joining method [31]. In addition, Illumina HiSeq2000 and Pacific Biosciences II sequencing platforms were used to sequence the whole genome of strain A17 [32] (Guangdong Meige Gene Technology Co., Ltd., Guangzhou, China). Relevant protein-coding genes were retrieved using GeneMarkS software (v.4.30) [33]. Prediction analysis of tRNA, rRNA and snRNA genes was performed by tRNAscan-SE, rRNAmmer, and Rfam databases [34,35]. The coding-protein genes were annotated with NR (NonRedundant Protein Sequence Database), KEGG (Kyoto Encyclopedia of Genes and Genomes), eggNog (Non-supervised Orthologous Groups) databases, and Swiss-Prot [36,37].

### 2.5. Physiological Parameters and Degradation of Petroleum Hydrocarbons by A17

A17 was grown in MSM-petroleum hydrocarbon liquid medium with 150 r/min shaking at different temperatures (25 °C, 30 °C, 37 °C, and 42 °C), pH (4, 5, 6, 7, 8, 9, 10, and 11) and salinity (2.0%, 4.0%, 6.0%, 8.0%, and 10.0%) [38]. The optical density (OD_600_) of the cultures was measured with a UV-vis spectrophotometer (DR3900, HACH, Loveland, CO, USA) at different time intervals: 0, 8, 28, 36, 48, 60, 72, and 96 h for varying temperatures and pH levels, and at 72 h for different salinities. The pH of the petroleum hydrocarbon-MSM liquid medium with HCl and NaOH to adjust. NaCl is used to regulate the salinity of the medium. The content of real-time metabolized petroleum hydrocarbons in the medium was determined by GC-MS. (QP2010, Shimadzu Instruments Manufacturing Co., Ltd., Kyoto, Japan), the detection procedure is the same as described in Section 2.3. The experiment was conducted in triplicates for each sampling point. Subsequently, the rate of petroleum hydrocarbon breakdown and degradation half-life by A17 were determined under the optimal conditions.

### 2.6. Fluorescein Diacetate (FDA) Hydrolase Assay

Fluorescein diacetate (FDA) hydrolytic activity is often used to indirectly measure microbial activity. FDA can be widely hydrolyzed by enzymes. The final product of the hydrolysis reaction is fluorescein, which has strong absorption in the visible range (490 nm), making it easy to measure by spectrophotometry [39]. FDA hydrolysis was determined by a modification of the procedure of Inbar et al. [40]. The substrate stock solution of FDA (C_24_H_16_O_7_, purchased from Chinese Sigma-Aldrich) was prepared by dissolving 0.2 g FDA powder in 200 mL of analytical grade acetone. The preparation method of the standard solution of fluorescein (C_24_H_14_O_6_, Sigma-Aldrich, Shanghai, China) is to dissolve it in phosphate-buffered solution with a concentration of 1.0 g L^−1^. To avoid degradation and concentration decrease, both solutions were stored in amber bottles in a refrigerator at 4 °C. After optimization, the final program is as follows: 1 g of fresh precipitate (bacterial solution centrifuged at room temperature and 6000 rpm for 5 min) was mixed with 15 mL of 50 mM phosphate-buffered solution (pH 7.3) in a 50 mL conical flask. Sterilized glass beads (2 g, diameter 0.5 mm) were slowly added to the mixture, followed by 0.3 mL of substrate stock solution. A control was set up using 0.3 mL of acetone instead of the substrate solution. The precipitate sample was incubated in a shaker (MD-M3R, Shanghai Minquan Instrument Co., Ltd., Shanghai, China) at 35 °C and 50 rpm for 1 h. Then, 2 mL of acetone was added to the precipitate aqueous matrix to terminate the reaction. The culture was then centrifuged at 5000 *g* for 5 min. The supernatant was filtered and transferred to a cuvette for measurement of absorbance at 490 nm with UV-vis spectrophotometer (DR3900, HACH, USA).

### 2.7. Degradation of Different Carbon-Chain Alkanes and Intermediate of Long-Chain Alkane Degradation by A17

Under optimal conditions, the degradation ability of strain A17 towards different carbon chain alkanes (C16, C20, C25, C29, and C35) was investigated using resting cells. Specifically, distinct carbon sources were prepared in MSM medium through the addition of alkanes (at a concentration of 500 mg/L) of C16, C20, C25, C29, and C35, respectively. Subsequently, A17 resting cell suspension (OD600 = 5.0) was introduced into each medium and cultured for three days. The growth of bacterial cells was quantified by UV spectrophotometry (DR3900, HACH, USA), with distilled water serving as a control for measuring OD600 values. The residual petroleum hydrocarbon content in the medium was determined by GC-MS (QP2010, Shimadzu Instruments Manufacturing Co., Ltd., Kyoto, Japan) analysis, with three parallel samples set up for each condition.

To detect intermediate products, n-C25 was supplemented into the MSM medium at pH 9.0, and the seed culture was inoculated into this medium and cultured at 30 °C with shaking at 150 r/min. Samples were collected periodically, and HCl was added to adjust the pH of the samples to 2.0 to terminate the reaction. Thereafter, samples were extracted with an equal volume of n-hexane, and 1 mol/L NaOH-CH_3_OH solution was added followed by shaking. After methylation at 50 °C in a water bath for 0.5 h, the samples were subjected to GC-MS analysis.

### 2.8. Antibiotic Resistance of Strain A17

A17 was grown to reach the exponential phase in 10 g/L MSM-petroleum hydrocarbon medium at pH 9.0. Cells were collected by centrifugation, washed twice with sterile 1× PBS, and diluted to an OD600 value of 0.5. Plate spreading was conducted with the cell suspension on LB medium. Antibiotic resistance test was conducted by using Oxford cups method [41] at 30 °C in an incubator (BSG-250, Shanghai Boxun Industrial Co., Ltd., Shanghai, China). The diameter of the inhibition zone was measured by a rule. The experiment was conducted in triplicates.

### 2.9. Statistical Analysis

Analyses of variances (ANOVA) was applied to evaluate the significant differences between samples and treatments using SPSS 27. statistical software. Statistically significant results were determined at *p* < 0.05. Curve fitting was performed according to the second-order kinetics equation [42] shown as follows:

(1) $$\frac{1}{C_{A}}-\frac{1}{C_{0}}=k_{A}t$$

where *C*_0_ represents the initial concentration of petroleum hydrocarbon (g/L); *C_A_* represents the final concentration of petroleum hydrocarbon (g/L); *k_A_* represents the reaction rate constant (Lg^−1^d^−1^) which is 0.0034 in our study; *t* represents the reaction time (d).

The half-life of petroleum degradation was calculated using the equation below [42]:

(2) $$T_{1/2}=1/k_{A}C_{0}$$

The kinetic model fitting was performed using time expressed in days. For clarity and consistency with the main text, degradation time and half-life values reported in the Results section were converted and presented in hours (h).

### 2.10. Access Number

The genome sequence of *Rhodococcus* sp. A17 (accession number PRJNA805410) was deposited in the NCBI database. The strain was tested for viability at the China General Microbiological Culture Collection Center (CGMCC) and stored in CGMCC under serial number MCGMCC No.23724.

## 3. Results and Discussion

### 3.1. Rhodococcus Rapidly Adapted to High Content of Petroleum at Alkaline pH

**Figure 1:**
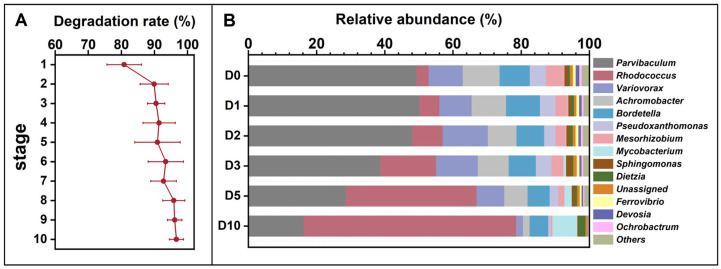
Petroleum hydrocarbon degradation rates across ten enrichment generations (**A**) and temporal changes in the relative abundance of the petroleum-degrading microbial consortium obtained after enrichment (**B**), cultivated in 1% petroleum hydrocarbon medium for 10 days and sampled at 0, 1, 3, 5, 7, and 10 days (corresponding to D0, D1, D3, D5, D7, and D10). The ten enrichment batches are designated as Stage 1 to Stage 10. Relative abundances in panel (**B**) do not directly correspond to Stage 1–10 in panel (**A**).

The microbial consortium from the indigenous soil microbial community of Dagang oilfield exhibited an increased degradation rate to petroleum hydrocarbon during the enrichment at alkaline pH (Figure 1A). The petroleum degradation rate reached 96.6% at the last round of the enrichment. The results indicate that (1) the indigenous soil microbial community has the potential to degrade petroleum. (2) during the enrichment process, several key microbial taxa potentially involved in petroleum degradation showed an increase in relative abundance. (3) the final petroleum hydrocarbon-degrading consortium was dominated by these taxa, suggesting an enrichment of functional groups. Microbial community profiling at genus level of the final consortium revealed that the seven most abundant genera were *Parvibaculum* (49.3%), *Rhodococcus* (3.5%), *Variovorax* (10.0%), *Achromobacter* (10.8%), *Bordetella* (8.8%), *Pseudoxanthomonas* (4.7%), and *Mesorhizobium* (5.3%) (Figure 1B). After 10 days of incubation in alkaline MSM medium, the relative abundance of Parvibaculum decreased to 16.2%, while Rhodococcus increased to 62.3%, representing a marked shift in community composition under these conditions. This temporal pattern indicates that *Rhodococcus* was selectively enriched under the applied incubation conditions and may play an important role during the later stages of petroleum hydrocarbon degradation in alkaline MSM medium. Members of the genus *Rhodococcus* are widely recognized for their metabolic versatility and capacity to degrade a broad spectrum of hydrophobic organic compounds, including alkanes and other petroleum-derived hydrocarbons, as reported in previous studies [43,44,45]. The observed enrichment of Rhodococcus under alkaline and petroleum-rich conditions in this study is therefore consistent with its known ecological and metabolic traits. The ability of *Rhodococcus* to persist and increase in abundance at high petroleum concentrations and elevated pH may be attributed to a combination of physiological characteristics, such as a structurally robust cell envelope, diverse catabolic pathways, and mechanisms for maintaining intracellular pH homeostasis [46]. Although these adaptive features were not directly quantified in the present study, they provide a plausible explanation for the competitive advantage of *Rhodococcus* during the enrichment process. These findings suggest that *Rhodococcus* spp. have potential for application in the bioremediation of petroleum contamination in alkaline pH environments [47].

### 3.2. Characteristics of Stain A17 Sustained Its Fast Acclimation to High Content of Petroleum at Alkaline pH

**Figure 2:**
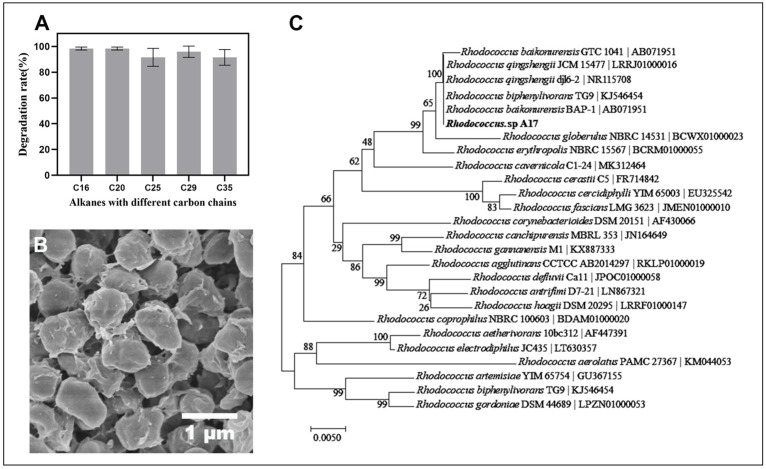
Degradation performance of *Rhodococcus* sp. A17 toward alkanes with different carbon chain lengths, together with its morphology and phylogenetic relationships. Panel (**A**) shows the degradation rates of strain A17 for individual alkanes. Panel (**B**) shows the cell morphology of strain A17 observed by scanning electron microscopy at 25,000× magnification. Panel (**C**) shows a phylogenetic tree of Rhodococcus sp. A17 and closely related species based on 16S rRNA gene sequence similarity, constructed using the Neighbor Joining method with the Tamura Nei genetic distance model. The analysis indicates that strain A17 shares 100% sequence similarity with several species within the genus *Rhodococcus*.

Strain A17, which isolated from the enrichment of the indigenous soil microbial community in Dagang oilfield and demonstrated a strong capacity to degrade high concentrations of long-chain petroleum hydrocarbons under alkaline conditions. The degradation rate for long-chain petroleum hydrocarbon was over 90% and did not show marked difference with the length of the carbon chain (*p* = 0.23) (Figure 2A). This observation is noteworthy, as previous research has shown that the degradation rate of petroleum hydrocarbons can vary based on the chain length, with longer chain lengths being more difficult to degrade [48]. Therefore, the result plausibly indicates, if not all, that strain A17 is well adapted to utilize long-chain petroleum hydrocarbons under alkaline conditions. The SEM observation of the cell morphology showed an oval shape with an apparent accumulation of extracellular material on the cell surface (Figure 2B). While the chemical nature of this material was not directly characterized, its presence is consistent with extracellular substances that have been reported in hydrocarbon-degrading bacteria. Previous studies have shown that extracellular polysaccharides can facilitate hydrocarbon biodegradation by enhancing bacterial adhesion to hydrophobic substrates, stabilizing microbial communities, and improving substrate accessibility [49,50]. In addition, extracellular polymers may function as biosurfactant-like substances, reducing the surface tension of the hydrocarbons and improving the access of the bacteria to the hydrocarbons for degradation [51]. However, further targeted analyses are required to confirm the composition and functional role of these extracellular materials in strain A17. Phylogenetic analysis based on the 16S rRNA gene sequence of strain A17 showed 100% similarity to several known species in the genus *Rhodococcus* (Figure 2C). Given the limited taxonomic resolution of the 16S rRNA gene within this genus, strain A17 was conservatively designated as *Rhodococcus* sp. A17 pending further taxonomic characterization.

**Figure 3:**
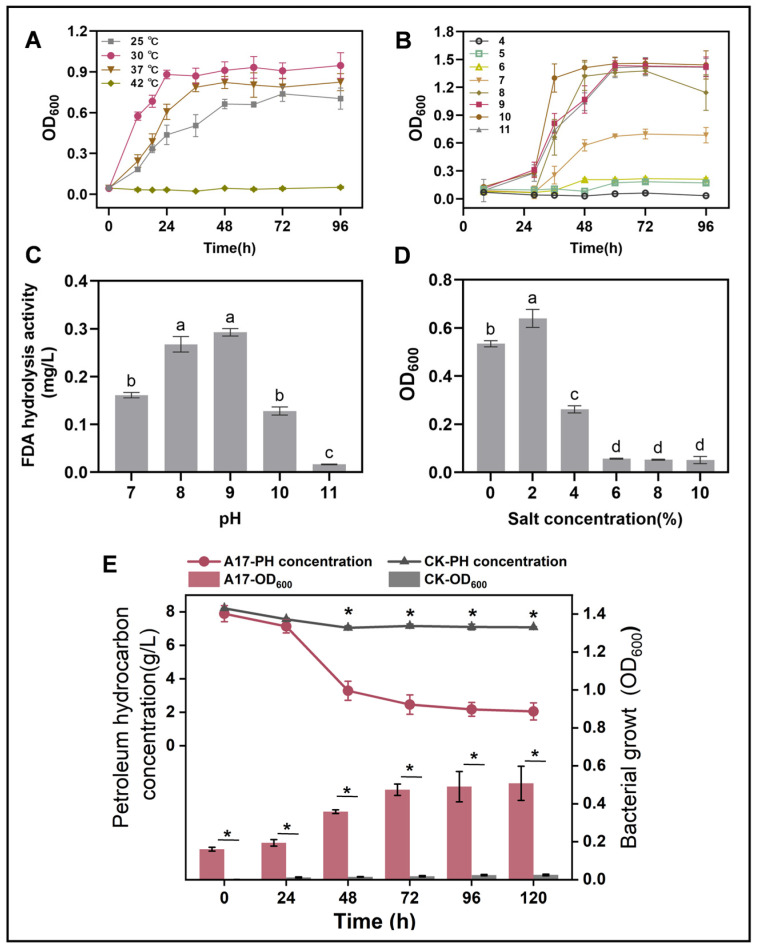
Growth performance of strain A17 under different environmental conditions (**A**–**D**) and its petroleum hydrocarbon degradation under the optimal growth conditions (**E**). Panel (**A**) shows the effects of temperature, panel (**B**) shows the effects of pH, panel (**C**) shows FDA hydrolytic enzyme activity of strain A17 under different pH conditions, and panel (**D**) shows the effects of salinity. Different lowercase letters indicate significant differences among treatments at *p* < 0.05. The optimal growth conditions were determined based on the combined effects of temperature, pH, and salinity. Panel (**E**) simultaneously presents microbial growth and petroleum hydrocarbon degradation measured at identical time points under the optimal conditions, allowing direct comparison of biomass increase and hydrocarbon removal during the degradation process. Asterisks indicate significant differences between two groups at *p* < 0.05. CK denotes the non-inoculated control, whereas A17 indicates the treatment inoculated with strain A17.

Our investigation uncovered that *Rhodococcus* sp. A17 was a mesophile, with an optimal growth temperature of 30 °C, and was unable to sustain growth at 42 °C. (Figure 3A). This result is in consistent with that of previous research that most species of *Rhodococcus* grow well between 20 and 45 °C, with optimal growth temperatures typically around 30–35 °C [52,53,54]. *Rhodococcus* sp. A17 exhibited optimal growth between pH 8 and 10, and FDA hydrolysis activity peaked at pH 9, without significant difference compared to pH 8 (Figure 3B,C). While most *Rhodococcus* species generally grow within pH 6–8, with optimal growth around pH 7–7.5 [55,56], strain A17 displayed a notable shift toward alkaline tolerance. Because NaOH was used to adjust the medium pH, sodium ions were introduced, suggesting that the observed growth and enzymatic activity at pH 8–10 may reflect combined effects of alkaline conditions and sodium availability rather than pH alone. Nevertheless, the ability of strain A17 to sustain growth and enzymatic activity under these conditions indicates physiological adaptation advantageous for petroleum-contaminated alkaline environments. In addition, strain A17 grew fastest in MSM-petroleum hydrocarbon liquid medium at 2% salinity and maintained substantial growth at 4% salinity (Figure 3D). This is consistent with previous reports that most *Rhodococcus* species are able to grow over a salinity range of 0–6% (*w*/*v*) NaCl, with optimal growth typically observed at lower salt concentrations [57,58]. The batch experiment of the kinetic of high pH petroleum degradation initiated by strain A17 revealed that the rate of petroleum hydrocarbon breakdown was 65.6% after 72 h incubation and the degradation half-life was determined as 34.2 h through a curve fitting analysis (Figure S1). In general, the degradation of petroleum compounds by *Rhodococcus* species can be quite rapid, with some studies reporting degradation rates on the order of several milligrams per liter per day [59,60].

### 3.3. Genomic Profiling Unveiled the Mechanisms of Petroleum Degradation Initiated by Rhodococcus sp. A17 at Alkaline pH

**Figure 4:**
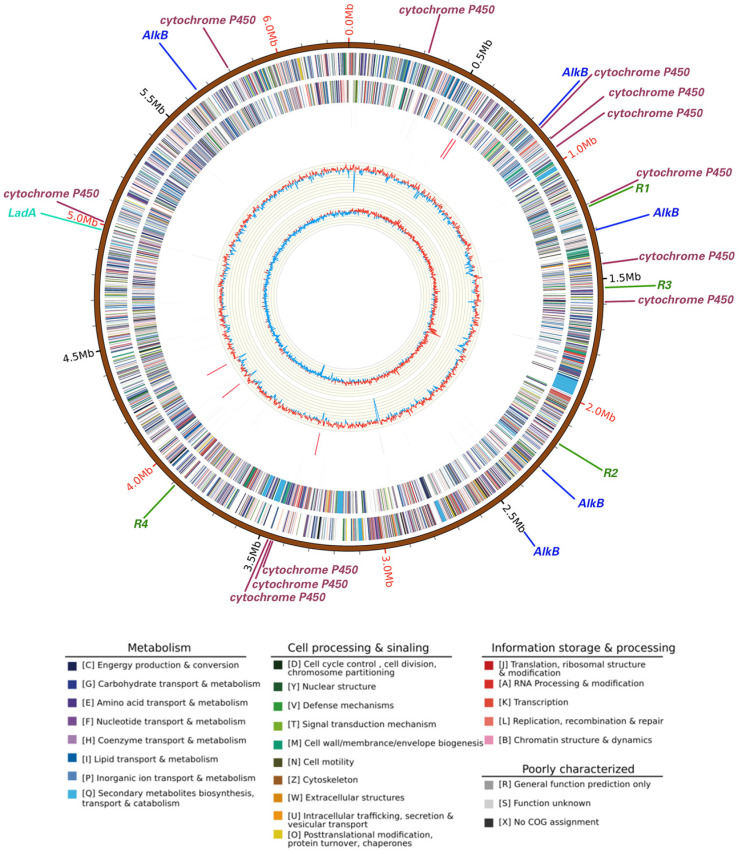
Circular map of the whole genome of *Rhodococcus* sp. A17. Rings from the outermost to the center represent the genome scale, protein coding genes on the forward strand, protein coding genes on the reverse strand, tRNA genes shown in black and rRNA genes shown in red on the forward strand, tRNA genes shown in black and rRNA genes shown in red on the reverse strand, GC content, and GC skew. Protein coding genes are color coded according to their COG functional categories. Straight lines outside the genome scale indicate the locations of petroleum hydrocarbon degradation genes and antibiotic resistance genes. Different colors represent different gene categories, while line length is not proportional to gene size and serves only to indicate gene position. Gene labels are shown at the end of each line, with R1, R2, R3, and R4 indicating four distinct antibiotic resistance genes.

The whole genome size of *Rhodococcus* sp. A17 was 6.71 Mbp in size (Figure 4) with an average GC content of 62.4%, comprising 6618 protein-coding genes, 15 rRNA operons, and 52 tRNA genes. The strain contained one circular chromosome (6,285,196 bp) and one stranded plasmid (433,169 bp), with an average GC content of 61.1%. Functional annotation of protein-coding genes revealed that the presence of multiple genes implicated in alkane degradation (Figure 4). Specifically, 18 genes encoding enzymes involved in the initial oxygenation steps of alkane degradation were identified, including the alkane monooxygenase *AlkB* [61], a family of cytochrome P450 family enzymes [62], and the long-chain alkane degrading enzyme *LadA* [63]. Five copies of *AlkB* were present, suggesting astong genetic potential for alkane hydroxylation [64], while 12 cytochrome P450 genes, particularly members of the CYP153 family, may contribute to the initial oxidation of alkanes [65]. Only one copy of the *LadA* gene was detected, encodes an enzyme capable of oxidize C15-C36 alkanes to the corresponding primary alcohols [63]. The catalytic activity of *LadA* follows a flavin-dependent monooxygenase mechanism, indicating that substrate binding governs its long-chain alkane degradation capability [66]. Although long-chain petroleum hydrocarbons are generally more difficult to degrade due to their size and chemical structure [67]. Strain A17 exhibited similar degradation rates across different chain lengths, suggesting the possible presence of additional metabolic pathways beyond *LadA*.

From the perspective of functional annotation, the alkane metabolic pathway starts with terminal and sub-terminal hydroxylation of alkanes to form alcohols [63], followed by dehydrogenase action to produce fatty acids and esters. To test this proposed metabolic pathway, the formation of intermediate products during n-C25 degradation was analyzed by GC–MS. At 0 days, only n-C25 was detected (peak at 53.5 min), with no additional peaks (Figure S2A). After 1 day, two new peaks appeared at 50.8 min and 54.7 min, corresponding to n-C23 acid and n-C25 acid, respectively (Figure S2B). By day 3, n-C25 was completely degraded, with no additional intermediates observed. These findings, combined with genome annotation of alkane-degrading enzymes, further confirm that n-C25 metabolism in strain A17 proceeds primarily via terminal and sub-terminal oxidation, producing fatty acids and esters that are further degraded [68].

Additionally, the genome of *Rhodococcus* sp. A17 contained four genes associated with antibiotic resistance. Consistent with the results shown in Table S1, the strain exhibited resistance to tetracycline, kanamycin, gentamicin, and chloramphenicol. Previous studies have shown that some strain exhibit a significant correlation between antibiotic resistance and hydrocarbon degradation ability [69]. The ability of microorganisms to degrade petroleum compounds and their resistance to antibiotics may be linked due to several reasons. Firstly, previous studies have reported that many petroleum-degrading microorganisms are exposed to high levels of pollutants, including antibiotics, in contaminated environments, which may select for microorganisms with evolved resistance mechanisms [70,71]. Secondly, it has been suggested that many enzymes used by microorganisms to degrade petroleum compounds, such as monooxygenases and cytochrome P450 enzymes, are similar to those used to degrade antibiotics, potentially conferring cross-resistance [65,72,73]. Finally, some of the pathways used by microorganisms to degrade petroleum compounds produce toxic intermediates that can be harmful to the microorganisms themselves [74]. As a result, microorganisms that are able to degrade petroleum compounds may have evolved mechanisms to resist antibiotics as a means of protecting themselves from these toxic intermediates.

## 4. Conclusions

In this study, an indigenous petroleum-degrading microbial consortium was enriched from alkaline petroleum-contaminated soil collected from the Dagang oilfield, China. From this consortium, *Rhodococcus* sp. A17 was successfully isolated and characterized as a petroleum-degrading bacterium capable of sustained growth under alkaline conditions. Physiological assays demonstrated that strain A17 exhibited optimal growth and hydrocarbon degradation activity at pH 9.0 and efficiently degraded long-chain petroleum hydrocarbons. Genome annotation revealed the presence of multiple genes associated with alkane oxidation and stress response, including alkane monooxygenases, cytochrome P450 enzymes, and long-chain alkane monooxygenase (*LadA*), providing a genetic basis that may contribute to hydrocarbon degradation under alkaline conditions. In addition, intermediate metabolite detection supported the involvement of both terminal and sub-terminal oxidation pathways during n-alkane degradation. Taken together, this experimental data, genomic analysis, and metabolite evidence suggest that several adaptive features could contribute to the ecological performance of strain A17 in petroleum-contaminated alkaline environments, including effective utilization of long-chain hydrocarbons and tolerance to alkaline stress. While the precise molecular mechanisms underlying these adaptations remain to be fully elucidated, the integrated findings offer a reasoned framework for understanding the biodegradation potential of Rhodococcus sp. A17. Overall, this study expands current knowledge of alkaliphilic petroleum-degrading Rhodococcus strains and highlights the potential applicability of strain A17 in the bioremediation of petroleum-contaminated environments with elevated pH, based on experimental observations.

## Data Availability

Dataset available on request from the authors.

## Supplementary Materials

The following supporting information can be downloaded at: https://www.mdpi.com/article/10.3390/life16020252/s1, Figure S1: Fitting curve of second-order reaction kinetics of petroleum hydrocarbon degradation; Figure S2: GC–MS analysis of characteristic intermediate metabolites during n-pentacosane degradation by strain A17; Table S1: Resistance detection of stain A17.
