# Supplementary material for: Molecular Insights into Rhodococcus sp. A17: Physiological Adaptations and Degradation Characteristics for Organic Contamination at Alkaline pH
Source: Life (Basel). 2026 Feb 2;16(2):252. doi: 10.3390/life16020252 (PMC12941523; doi:10.3390/life16020252)
Supplement: Supplementary file 1 [file life-16-00252-s001.zip › life-4092609-supplementary.pdf]

Molecular Insights into *Rhodococcus sp.* A17: Physiological Adaptations and Degradation Characteristics for Petroleum Contamination at Alkaline pH

Xinyuan Wei[1, 2, 3, †], Haoyu Wang[1, †], Rui Li[1, 2], Shengmin Liu[4], Hongyan Zuo[5], Qing Hu[3, 5*], Xuliang Zhuang[1, 2, 6], Zhihui Bai[1, 2, 3, *]

[1] Research Center for Eco-Environmental Sciences, Chinese Academy of Sciences, Beijing 100085, China; weixinyuan20@163.com (X.W.); bqt1800302025@student.cumtb.edu.cn (H.W.); ruili_st@rcees.ac.cn (R.L.); zhbai@rcees.ac.cn (Z.B.); xlzhuang@rcees.ac.cn (X.Z.)
[2] University of Chinese Academy of Sciences, Beijing 100049, China
[3] Xiong'an Institute of Innovation, Chinese Academy of Sciences, Baoding 071700, China
[4] Dingzhou Fuyuan Food Co., Ltd, Dingzhou 073000, China
[5] State Key Laboratory of Lunar and Planetary Sciences, Macau University of Science and Technology, Taipa 999078, Macau
[6] Institute of Tibetan Plateau Research, Chinese Academy of Sciences, Beijing 100101, China
* Correspondence: zhbai@rcees.edu.cn (Z.B.); huqing@xii.ac.cn (Q.H.); Tel.: +86-10-6284-9156 (Z.B.); +853-6855-7877 (Q.H.)

† These authors contributed equally to this work.

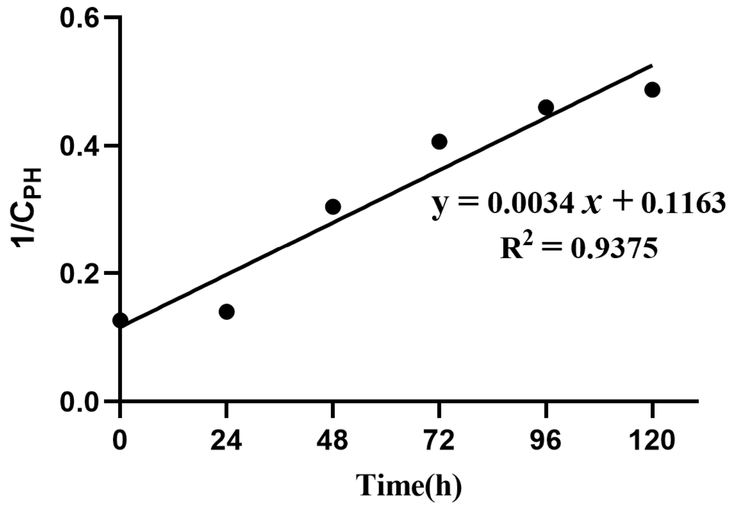

Figure. S1. Fitting curve of second-order reaction kinetics of petroleum hydrocarbon degradation. The C_{PH} in the vertical coordinate represents the final concentration of petroleum hydrocarbon (g/L).

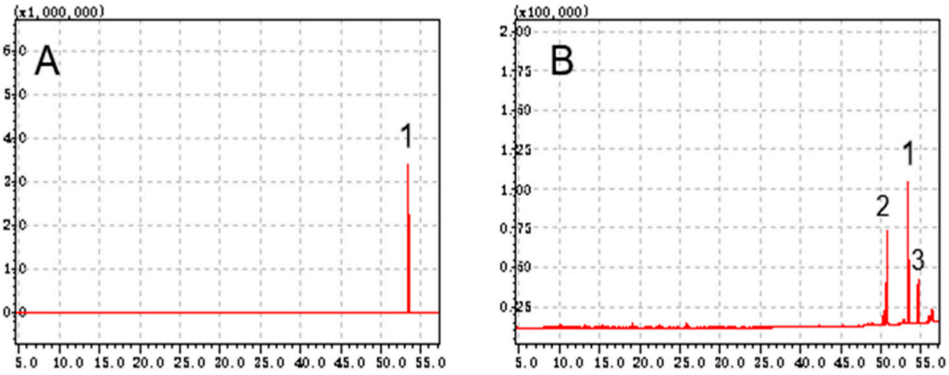

Figure. S2. GC–MS analysis of characteristic intermediate metabolites during n-pentacosane degradation by strain A17. At 0 days, only peak 1 (A), corresponding to n-pentacosane, was detected at 53.5 min. After 1 day of incubation, two new peaks, 2 and 3 (B), appeared at 50.8 min and 54.7 min, corresponding to n-tricosanoic acid and n-pentacosanoic acid, respectively.

Table S1. Resistance detection of stain A17. The values are presented in the average ± standard deviation (SD, n=3). R: resistance(＜1cm), I: intermediary(1-2cm), S: sensitivity(＞2cm).

Strain	Antibiotic	Average diameter of bacteriostatic circle （cm）	Degree
A17	Tetracycline	3.18±0.07	S
	Kanamycin	2.83±0.03	S
	Gentamicin	4.42±0.01	S
	Chloramphenicol	0.82±0.02	R
